# Complete response and long-term survival to endocrine monotherapy in a patient with metastatic breast cancer in a low-income country: a case report

**DOI:** 10.1186/s13256-024-04734-6

**Published:** 2024-09-04

**Authors:** Raghad Sada, Nouralhuda Karim, Ghina Rohaibani, Mousa Alali, Maher Saifo

**Affiliations:** 1 Faculty of Medicine, Damascus University, Fayez Mansour Street, P. O. Box: 222, Damascus, Syrian Arab Republic; 2 Department of Oncology, Albairouni University Hospital, Faculty of Medicine, Damascus University, Harasta M5, Damascus, Syrian Arab Republic

**Keywords:** Survival, Endocrine therapy, Breast cancer, Malignant pleural effusion

## Abstract

**Background:**

Breast cancer is the most common cancer in women. Progression-free survival for hormone receptor-positive/human epidermal growth factor receptor-2-negative metastatic breast cancer treated with endocrine therapy in combination with cyclin4/6-dependent kinase is approximately 25 months. This case represents metastatic breast cancer treated with endocrine therapy, leading to long-term survival.

**Case presentation:**

A 40-year-old Syrian woman diagnosed with hormone receptor-negative breast cancer was treated surgically with adjuvant chemotherapy and radiotherapy. She developed local and nodal recurrences that were hormone receptor-positive, followed by a recurrence of malignant pleural effusion. She was initially treated with chemotherapy and then placed on endocrine therapy with a complete response from 2014 until now. The patient also suffered from adverse events of medications, such as heart failure and osteoporosis, which were treated appropriately.

**Conclusion:**

This case demonstrates a long-lasting complete response to metastatic breast cancer with malignant pleural effusion. This shows the validity of endocrine therapy in recurrent hormone receptor-positive breast cancer, especially in countries that cannot afford targeted therapies or genetic tests. It also highlights the necessity for a better understanding of the prognostic and predictive factors.

## Background

Female breast cancer (BC) was the most commonly diagnosed cancer in 2020 (11.7% of total cases), and it caused 6.9% of the total cancer deaths in the same year and was the leading cause of cancer-related death in women [1]. Invasive ductal carcinoma (IDC) is the most common histological subtype of BC, accounting for 80% of BC [2]. Until now, metastatic breast cancer (MBC) is still incurable even after the development of new drugs. Progression-free survival (PFS) for hormone receptor (HR)-positive/human epidermal growth factor receptor-2 (HER-2)-negative MBC treated with the recommended first-line treatment is approximately 25 months [3]. Malignant pleural effusion (MPE) is a serious metastatic site of advanced malignant tumors [4]. This case represents MBC with MPE treated with endocrine therapy (ET) alone, leading to complete response (CR) and long-term survival (LTS).

## Case presentation

A 40-year-old Syrian premenopausal woman, who is married and has three children born vaginally with menarche at 13 years. She has no significant medical history, except that her father died because of lung cancer, and her niece suffered from BC. When the patient visited Albairouni University Hospital in September 2005, she had undergone a right partial mastectomy with axillary dissection; pathology showed a grade 1 IDC, HRs were negative, and HER-2 was 2+. The patient subsequently received adjuvant chemotherapy (ACT) with six cycles of 5-fluorouracil, doxorubicin, and cyclophosphamide (FAC), then radiation therapy. The patient did well and was very adherent to clinical and radiological follow-up.

In August 2009, she complained of a change in the right breast size, which was smaller with hardening of the retro-areolar part and nipple retraction. A clinical examination showed a small mass in her right breast and an enlargement of her right axillary lymph nodes. A biopsy of the lump showed IDC. Therefore, she received neoadjuvant chemotherapy with one cycle of FAC and two cycles of 5-fluorouracil and cyclophosphamide [the cumulative dose of doxorubicin is 630 mg (370 mg/m^2^)], followed by modified radical mastectomy with axillary dissection in November 2009. Pathological studies revealed a grade 2 IDC showing a focal tubular pattern, and axillary lymph nodes showed metastatic carcinoma in 2 out of 14 lymph nodes. HRs were negative, HER-2 was 2+, and the TNM stage was ypT1, N1, M0. After that, the patient was treated with ACT with six cycles of paclitaxel and carboplatin. Mild grade 1 neutropenia and anemia were recorded but did not require any dose modification. A subsequent chest/abdomen computed tomography (CT) and bone scan showed no signs of other metastasis. Then, the patient underwent clinical and radiological follow-up.

In November 2011, after clinical suspicion, the ultrasound showed a right supraclavicular nodule measuring 3.3 × 1.5 mm. Excisional biopsy revealed IDC. Immunohistochemistry showed expression of progesterone receptor (70%) and estrogen receptor (10%), and HER-2 status was 2+ (equivocal). The patient then underwent chemotherapy treatment with six cycles of paclitaxel followed by ET with tamoxifen.

In October 2012, a chest X-ray (CXR) and CT showed a bilateral pleural effusion with two nodules in the upper and lower lobes of the right lung. MBC was established from the finding of breathlessness accompanied by pleural effusion with cytology examination revealing MPE; therefore, the patient then underwent chemotherapy with carboplatin and intravenous vinorelbine. In January 2013, the patient was hospitalized for chemotherapy-induced cardiomyopathy with reduced ejection fraction (EF) of 45%. Therefore, carboplatin was stopped and replaced by capecitabine with oral vinorelbine until the end of 2013.

**Fig. 1:**
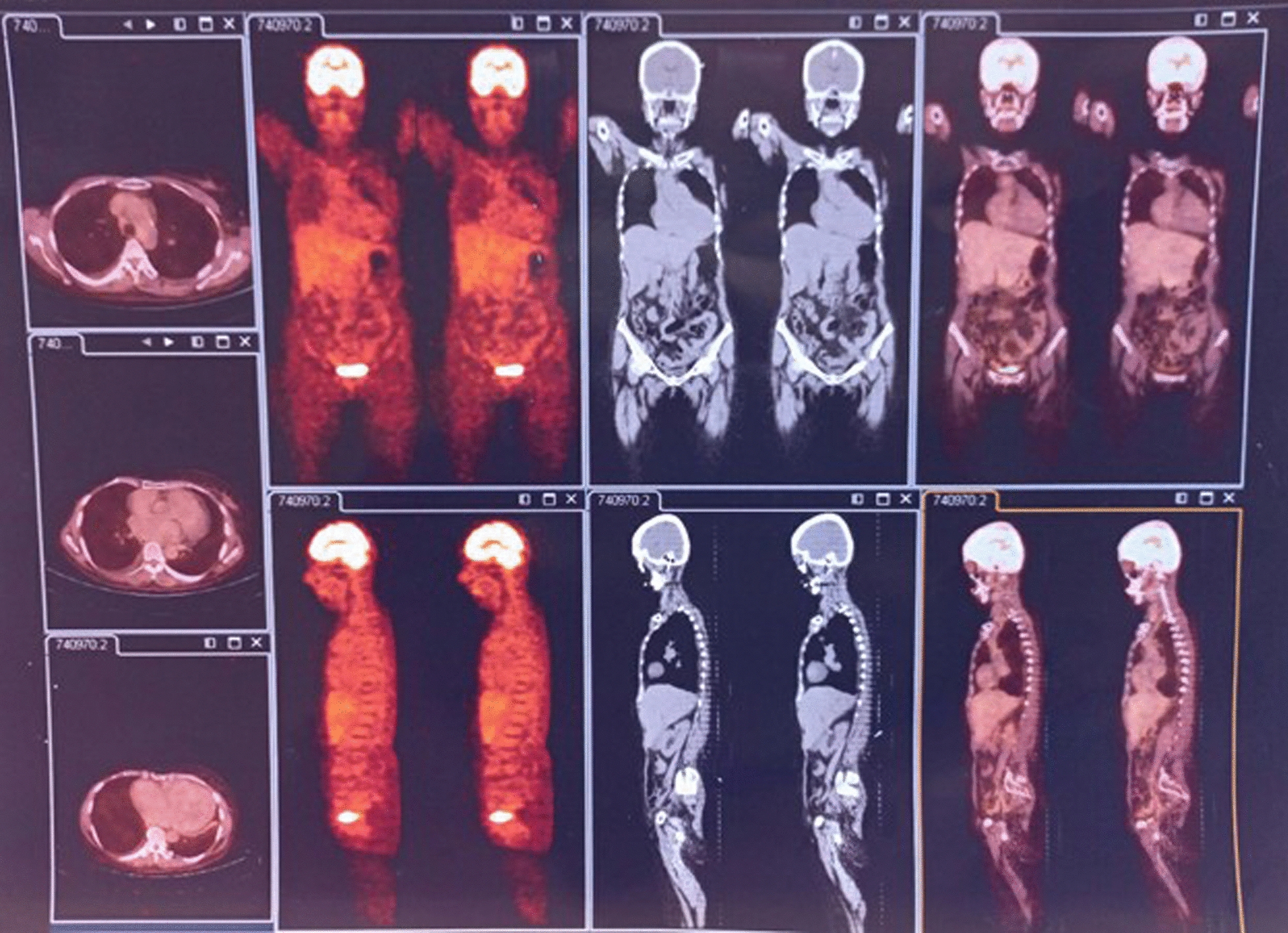
A positron emission tomography-computed tomography in October 2023 demonstrated a complete response to endocrine monotherapy

In January 2014, ET with aromatase inhibitors (AI) (anastrozole or letrozole as available) in combination with zoledronic acid periodically were scheduled for the patient. Clinical examinations were performed every 3 months, CXR, abdominal/pelvic ultrasound, laboratory tests, and tumor biomarkers every 6 months with CT and bone scan when required. The result was a complete response (CR). In 2021, osteoporosis was developed, and AI was replaced by tamoxifen. Currently, the patient complained of paroxysmal nocturnal dyspnea, CXR revealed interstitial pulmonary edema related to heart failure (EF: 33%), which was treated appropriately by a cardiologist. A positron emission tomography-CT in October 2023 demonstrated a CR to ET (Fig. 1). At this time, the patient continues to respond to the current therapy and is doing well.

**Fig. 2:**
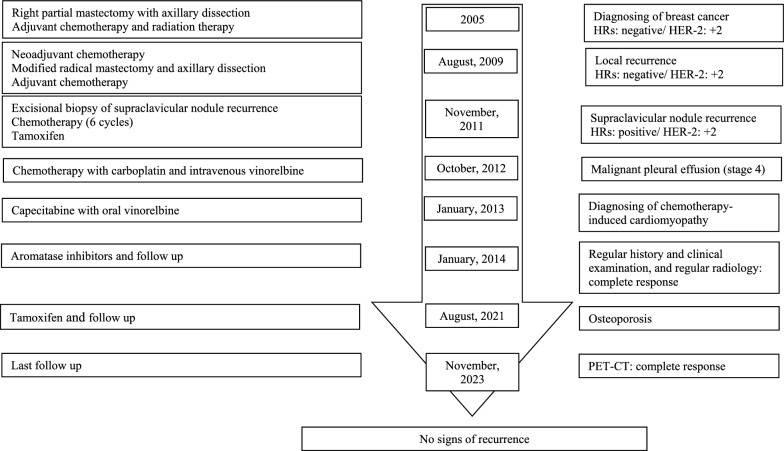
The timeline for the patient’s case. *HER-2* Human epidermal growth factor receptor-2, *HR* Hormone receptor, *PET-CT* Positron emission tomography-computed tomography

Since the patient will have completed 10 years of ET, her case will be presented to a multidisciplinary team at Albairouni University Hospital to discuss stopping treatment and keeping the patient on close follow-up. The timeline for the patient’s case is illustrated in Fig. 2.

## Discussion and conclusion

BC is the second leading cause of MPE after lung cancer. Approximately 7–11% of patients developed MPE during their disease history, which exhibits rapid progression and is associated with a poor prognosis [5]. Previous studies have demonstrated that patients with MPE have a median OS of only 3–12 months [6]. This case represents MBC treated with ET alone, leading to LTS.

The latest updated treatment guidelines of The National Comprehensive Cancer Network reported a combination of AI and cyclin4/6-dependent kinase inhibitors (CDKIs) as the preferred first-line therapy for HR-positive/HER-2-negative BC [7]. CDKIs (ribociclib) in combination with AI (letrozole) were studied as first-line therapy in a phase III study of postmenopausal patients (*n* = 668) with HR-positive/HER-2-negative recurrent/stage IV BC. At a median follow-up of 26.4 months, an improvement in PFS (25.3 versus 16.0 months; hazard ratio was 0.56, 95% confidence interval 0.45–0.70) and improved overall response rate of 43% versus 29% was seen with ribociclib plus letrozole compared with letrozole alone [3].

According to recent recommendations, our patient should have been treated with AIs and CDKIs, but CDKIs were not available, so she was treated with AIs alone [7]. Although the patient was treated with AI alone, she survived for ~ 135 months and is still alive.

Anastrozole and letrozole were given to the patient as available. As randomized controlled trials showed, there were no significant differences in the quality-of-life between the two drugs, and they had the same effectiveness [8]. After treating the patient with AIs for several years, she developed osteoporosis, so the AIs were replaced with tamoxifen, which has bone-protective effects in postmenopausal women [9].

A report published in 2020, like our case, is about a 39-year-old woman who was diagnosed with HR-positive/HER-2-negative BC with synchronous bone metastases, who experienced a disease response of 144 months with ET as maintenance after first-line chemotherapy with a good toxicity profile [10]. Additionally, Pahouja *et al*. [11] reported a patient with total bone marrow failure caused by MBC who was treated with doxorubicin followed by AIs, the response was good, with overall survival (OS) of 44 months [11]. In addition, in the case series by Freyer *et al*. [12] like the above, five patients were treated with ET, bisphosphonate, and low-dose chemotherapy, giving good outcomes with OS between 12 and 38 months [12].

This study showed discrepancies in the results of HRs and HER-2 between the primary tumor and recurrence, consistent with previous studies. Francis *et al*. [13] reported a difference in 63% of cases and highlighted the necessity of performing tissue biopsies to confirm the status of molecular markers when recurrence occurs.

Although the cumulative dose of doxorubicin in this patient was less than 400 mg/m^2^, the patient developed heart failure. Previous studies reported that the incidence of heart failure in these cases is 3–5% and 18–48% at 700 mg/m^2^, which confirms the necessity of periodic monitoring of heart function after receiving doxorubicin, especially when radiation therapy to the left chest wall is given [14].

One of the limitations of this report was that fluorescence in-situ hybridization was not available for the diagnosis in Albairouni University Hospital, so she was considered HER-2-negative. However, most HER-2 2 + (equivocal) BC cases were reclassified as HER-2-negative [15]. Moreover, there is no difference in prognosis according to the in-situ hybridization status in HER-2 2 + cases. Recent studies concluded that HER-2 2 + had unique biological characteristics from HER-2 negative or positive cases [16].

In conclusion, this case shows MBC treated with ET with CR, LTS, and good quality-of-life. It confirms that ET remains the cornerstone of treatment for MBC, especially in the absence of targeted agents. It also notes the long-term complications of oncogenic treatments. This case also demonstrates the challenges facing low-resource countries in cancer management.

## Data Availability

The data that support the findings of this study are available from the corresponding author upon reasonable request.

## Abbreviations

ACT: Adjuvant chemotherapy
AI: Aromatase inhibitor
BC: Breast cancer
CDKI: Cyclin4/6-dependent kinase inhibitor
CT: Computed tomography
CR: Complete response
CXR: Chest X-ray
EF: Ejection fraction
ET: Endocrine therapy
FAC: 5-Fluorouracil, doxorubicin, and cyclophosphamide
HER-2: Human epidermal growth factor receptor-2
HR: Hormone receptor
IDC: Invasive ductal carcinoma
LTS: Long-term survival
MBC: Metastatic breast cancer
MPE: Malignant pleural effusion
OS: Overall survival
PFS: Progression-free survival
